# Giant Splenic Pseudocyst in an Adult Patient: A Case Report

**DOI:** 10.7759/cureus.89620

**Published:** 2025-08-08

**Authors:** Sergio G Moreno Hernandez, Cristina Grado Limas, Luis Cruz Benitez, Rosa M Morales López, Luis Díaz Hernández, Omar E Valencia-Ledezma, Pedro J Curi-Curi, Arath A Zamarripa Olmedo

**Affiliations:** 1 Surgery, Hospital Regional de Alta Especialidad de Ixtapaluca, Mexico City, MEX; 2 Surgery, Universidad Nacional Autónoma de México, Mexico City, MEX; 3 Pathology, Hospital Regional de Alta Especialidad de Ixtapaluca, Mexico City, MEX; 4 Research Unit, Hospital Regional de Alta Especialidad de Ixtapaluca, Mexico City, MEX; 5 Investigation, Hospital Regional de Alta Especialidad de Ixtapaluca, Mexico City, MEX

**Keywords:** chronic pain, cyst, laparoscopic, splenectomy, splenic pseudocyst

## Abstract

Splenic pseudocysts represent an uncommon condition in abdominal surgery, generally resulting from trauma, infection, or ischemic processes. Histologically, they are characterized by the absence of an epithelial lining; that is, they lack the inner layer of cells typically found in true cysts. Its clinical presentation is non-specific, commonly manifesting with abdominal pain, early satiety, or alterations in bowel habits, which lead to late or incidental diagnoses through imaging studies. We present a case of a 40-year-old male patient with chronic digestive symptoms of three years' evolution. After multiple medical consultations without a definitive diagnosis, a CT scan was performed that revealed a giant splenic cystic lesion measuring 15 × 20 cm, with a compressive effect on adjacent abdominal structures. Laparoscopic total splenectomy was indicated, preceded by pneumococcal and meningococcal vaccination. The intervention was performed without complications, and the patient was discharged 24 hours later with a satisfactory clinical evolution. The macroscopic study of the spleen showed a 20 x 15 cm unilocular cystic lesion with necrotic content, and histopathological analysis confirmed the diagnosis of splenic pseudocyst. This case was compared with a similar report in the literature, where a patient with a comparable lesion was treated by open splenectomy, also obtaining a favorable result. Both cases highlight the importance of maintaining a high index of suspicion in the face of non-specific abdominal symptoms and the usefulness of imaging as a diagnostic tool. They also reinforce the role of splenectomy, especially laparoscopically, as a safe and effective treatment in large lesions, always under a multidisciplinary approach and with adequate preventive immunological management.

## Introduction

Splenic cysts represent an uncommon clinical entity, with a significantly low prevalence within abdominal pathology. Within this category, splenic pseudocysts constitute an even rarer subclass, histologically characterized by the absence of an epithelial lining, which differentiates them from primary or true splenic cysts [1]. Their etiology is often associated with a history of splenic trauma, infectious processes, infarction, or tissue degeneration; however, in many cases, no clear triggering factor can be identified [2].

The clinical presentation of splenic pseudocysts is variable and, in most cases, nonspecific [3]. Patients may present with diffuse abdominal pain, postprandial fullness, distension, nonspecific digestive symptoms, or may even remain asymptomatic for prolonged periods [4]. This clinical nonspecificity, combined with the rarity of the entity, often delays diagnosis, which is frequently made incidentally through imaging studies performed for other reasons [5].

Computed tomography (CT) and magnetic resonance imaging (MRI) are key tools for identifying and characterizing these lesions, allowing evaluation of their size, contents, borders, and compressive effects on adjacent structures [6]. The management of splenic pseudocysts depends on multiple factors, such as size, symptomatology, location, and potential complications. Small, asymptomatic lesions can be managed conservatively with periodic follow-up. However, large, symptomatic pseudocysts or those at risk of rupture require surgical intervention [2].

Non-traumatic splenic pseudocyst is an extremely rare entity, characterized by the absence of epithelium in its wall. It is generally associated with trauma, so its occurrence without an apparent cause represents a diagnostic challenge [7]. Splenic cysts constitute an uncommon entity with variable clinical evolution. Comparison between cases highlights key differences in their etiology, presentation, and therapeutic approach [8].

In this context, total laparoscopic splenectomy has become a safe and effective option, offering the benefits associated with minimally invasive surgery, such as reduced postoperative pain, faster recovery, and improved aesthetic outcomes.

This report presents a case of an adult male patient with a giant splenic pseudocyst, whose prolonged clinical course and nonspecific digestive symptoms led to a delayed diagnosis. Through imaging evaluation, multidisciplinary management, and surgical resolution via laparoscopic splenectomy, a favorable outcome was achieved. This case underscores the importance of maintaining a high index of suspicion in atypical clinical presentations and highlights the role of timely diagnosis and appropriate management [9].

## Case presentation

A 40-year-old male patient with no relevant chronic-degenerative medical history presented with a three-year history of altered bowel habits, including episodes of diarrhea, epigastric abdominal pain, and early satiety. He consulted multiple physicians without obtaining a definitive diagnosis or symptom relief. He was referred to our hospital on August 2, 2024, and evaluated by the gastroenterology service following a non-contrast CT scan that reported a calcified splenic cyst measuring 1,364 cc.

**Figure 1:**
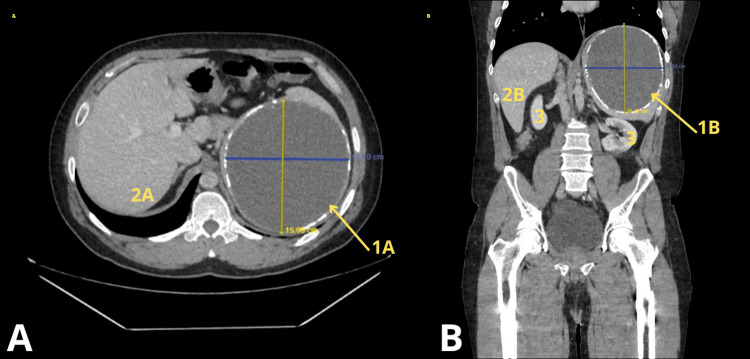
Computed tomography. Axial (A) and coronal (B) abdominal CT images demonstrating a primary splenic cyst. 1A: Axial view showing the splenic cyst. 2B: Coronal view showing the splenic cyst. 2A: Axial view of the liver. 2B: Coronal view of the liver. 3: Coronal view showing the right and left kidneys.

The patient was subsequently referred to our service on October 24, 2024. Physical examination revealed a soft but asymmetric abdomen due to abdominal distension predominantly in the epigastric region and a palpable, tender mass in the left hypochondrium. A new contrast-enhanced abdominal CT scan was requested (Figure 1). Imaging revealed an ovoid splenic lesion with well-defined, hyperdense (calcified) margins and homogeneous, hypodense content (21 HU), with no changes after intravenous contrast administration. The lesion measured 13.5 x 14.9 x 14.7 cm, with an estimated volume of 1,546 cc. It occupied the entire upper third of the spleen, exerting a mass effect on adjacent structures; inferior displacement of the left kidney, which appeared malrotated with a horizontal axis; anterior displacement of the pancreatic body and tail; and medial displacement of the stomach.

Surgical management with total laparoscopic splenectomy was proposed after administration of pneumococcal and meningococcal vaccines.

The procedure was performed using the standard technique employed at Hospital Regional de Alta Especialidad de Ixtapaluca (HRAEI), utilizing three ports: two working ports (12 mm and 5 mm) and one optical port (12 mm). The patient was positioned in the right lateral decubitus position, with lateral flexion of the surgical table to open the costophrenic space.

Intraoperative findings included a deformed spleen measuring approximately 30 x 15 cm, with dense adhesions to the transverse colon, left paracolic gutter, and diaphragm. Vascular hilar control was achieved with titanium endoclips (artery and vein ligated separately). The surgical specimen was extracted through an extended incision at the optical trocar site, at the umbilical scar, and sent for definitive pathological analysis. Fluid from the cystic lesion was also obtained for culture, which yielded no growth of aerobic bacteria, anaerobes, or fungi.

Preoperative and postoperative laboratory tests were compared with those obtained 12 hours after surgery (Table 1).

**Table 1:** Laboratory results.

Test	Preoperative	Postoperative	Reference value
Hemoglobin	16.1 g/dL	11.7 g/dL	13.8 - 18.5
Leukocytes	6.0/µL	10.3 /µL	3.84 - 9.79
Neutrophils	62.3%	67.3%	39.6 - 76.1
Platelets	191/µL	211/µL	147 - 384

The patient showed favorable postoperative progress, which led the medical team to decide on hospital discharge 24 hours after the surgical procedure. He was prescribed an oral prophylactic antibiotic regimen along with analgesics. Prior to discharge, he was informed of warning signs to monitor and was scheduled for follow-up in the outpatient clinic. At his next appointment, he was evaluated and had no complications, indicating a satisfactory postoperative course.

A definitive diagnosis of splenic pseudocyst was made.

Pathology report

Gross Examination

**Figure 2:**
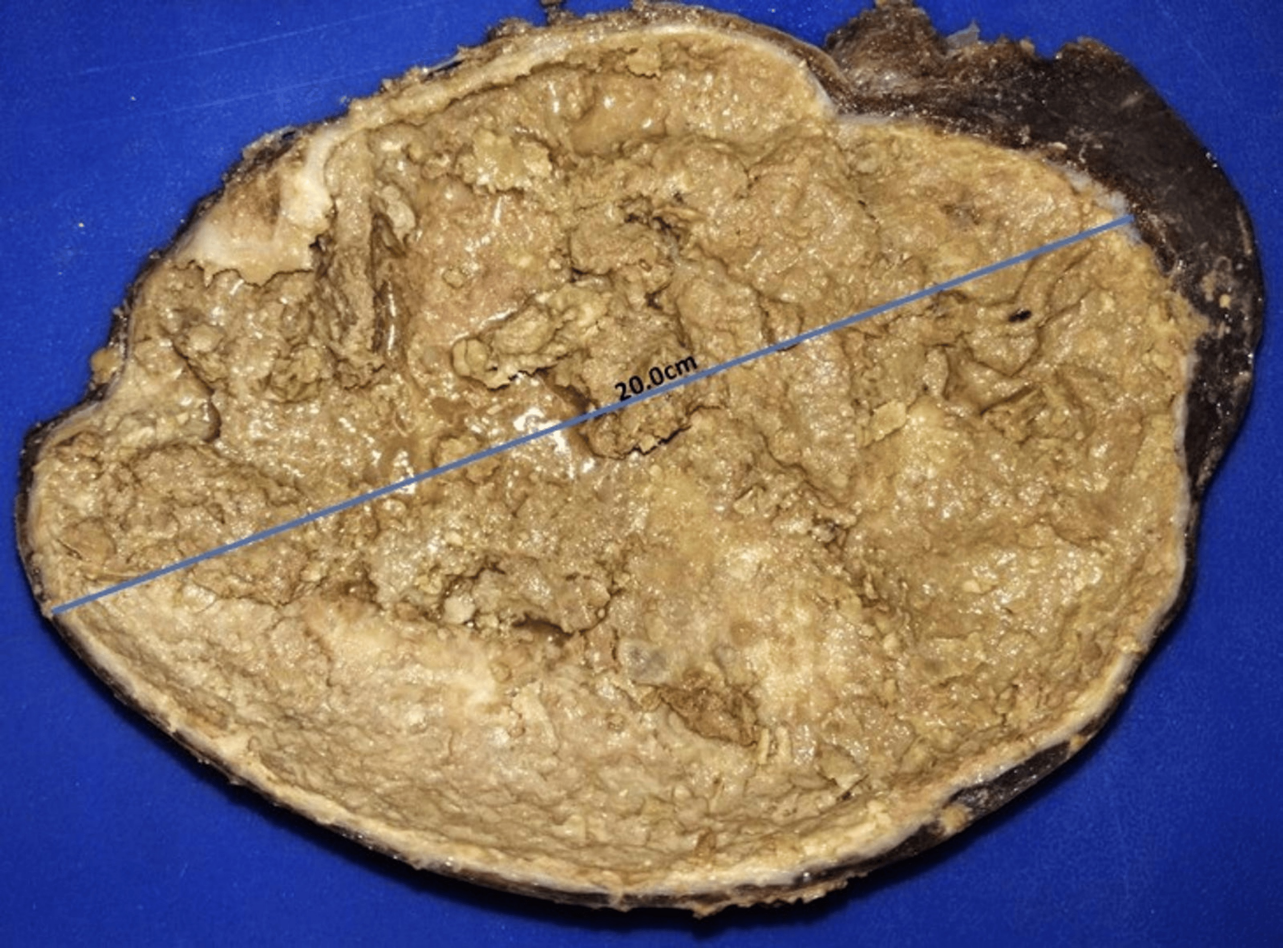
Macroscopic pathology image. Splenic pseudocyst (macroscopic view) measuring 20.0 cm in diameter.

The specimen was received in a formalin container labeled with the patient’s information and identified as “spleen,” the product of a splenectomy. The specimen weighed 655 g and measured 22.0 × 15.0 cm. It was grayish-brown in color with irregular, ovoid borders. On sectioning, the consistency was semi-firm with a heterogeneous cut surface, revealing a unilocular lesion measuring 20.0 × 15.0 cm. The cyst wall measured 0.3 cm in thickness and contained grayish, friable, necrotic-appearing material. Representative sections were submitted for histopathological examination in five cassettes (Figure 2).

Microscopic Examination

**Figure 3:**
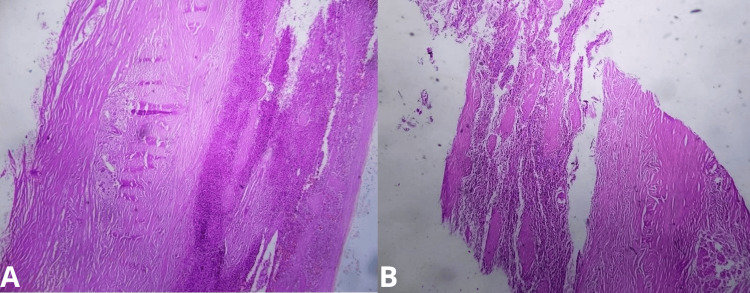
Microscopic pathology image. Images (A) and (B) show cyst wall fragments with fibrous tissue and absence of epithelial lining, consistent with the diagnosis of a pseudocyst.

Sections stained with hematoxylin and eosin revealed a cystic lesion without epithelial lining. The cyst wall was composed of dense fibrous tissue with calcifications, chronic inflammatory infiltrate, and necrotic debris (Figure 3).

## Discussion

Splenic pseudocysts are rare entities within benign splenic pathology. They are histologically defined by the absence of an epithelial lining and are generally associated with a history of trauma, infections, or ischemic events. Their clinical presentation is often nonspecific, with vague or chronic abdominal symptoms, and the diagnosis is frequently incidental through imaging studies performed for other reasons [10].

In the present case, no history of abdominal trauma was identified, suggesting alternative etiologies. Subclinical splenic infarctions or prior infections have been proposed as potential triggers of inflammatory processes that may eventually lead to the formation of splenic pseudocysts. These theories are supported in the literature as plausible explanations in cases where no direct trauma is documented. In this patient, a giant splenic pseudocyst was diagnosed by CT and successfully treated with laparoscopic splenectomy. The absence of postoperative complications and the patient’s rapid recovery reinforce the safety of the minimally invasive approach for large-volume splenic lesions [10].

This case was compared with multiple reports highlighting both clinical similarities and relevant differences. Navarrete et al. [10] reported a case of an immunocompetent adolescent female with a splenic cyst superinfected by *Salmonella* serotype B, which progressed to a ruptured abscess with hemodynamic compromise, managed with urgent splenectomy. In contrast to the present case, the clinical course was acute and of infectious origin, and the lesion was a primary epithelial cyst.

Karbasian et al. [11] documented a large non-parasitic splenic pseudocyst in an adolescent female with similar symptomatology and surgical management; however, an open splenectomy was performed due to the lesion’s size and compressive effect. Similarly, Salamanca et al. [12] described a young female with nonspecific symptoms and comparable CT findings, who also underwent laparoscopic splenectomy. Histopathology confirmed the absence of epithelial lining, the presence of calcifications, and necrotic contents. Nevertheless, relevant differences were observed in lesion size, clinical evolution, and intraoperative findings, such as the presence of an accessory spleen and absence of adhesions in the Colombian case [12].

Alhaddad et al. [13] reported a case very similar to ours in a 56-year-old male without a clear history of trauma, who presented with vague abdominal symptoms and an incidental diagnosis of a large-volume pseudocyst with calcified walls. Surgical management was identical: total splenectomy preceded by vaccination, with a favorable postoperative course.

In contrast, Abu Sabha et al. [14] described a conservative approach in a young male patient with a giant splenic pseudocyst, opting for aspiration of the cyst contents and partial cystectomy. This strategy aimed to preserve splenic immune function, representing an emerging trend in selected cases, although its application remains limited in large or centrally located lesions.

Across the reviewed cases, there is consensus regarding the diagnostic value of CT and the role of histopathology in differentiating pseudocysts from true cysts and parasitic lesions. Laparoscopy has also been confirmed as a safe and effective technique even in complex cases, provided the surgical team has appropriate expertise [12-14].

Finally, although the debate between total and partial splenectomy continues, evidence supports that with adequate preoperative vaccination, the risk of post-splenectomy sepsis is low. Therefore, in symptomatic patients or those with giant pseudocysts, as in the case presented, laparoscopic splenectomy emerges as an effective and safe therapeutic option [14].

## Conclusions

Splenic pseudocysts are uncommon and often represent a diagnostic challenge due to the nonspecificity of their symptoms and delayed recognition. This case highlights the importance of maintaining a high index of suspicion in patients with chronic abdominal complaints and the crucial role of imaging in reaching a timely diagnosis. While conservative measures such as aspiration or partial splenectomy may be appropriate in select cases, the presence of a large lesion (>15 cm), dense adhesions, and the risk of recurrence warranted a total laparoscopic splenectomy in this patient. Preoperative pneumococcal immunization against encapsulated organisms was administered according to current guidelines to minimize the risk of post-splenectomy infection, although the lack of long-term immunological follow-up remains a limitation. This case supports total laparoscopic splenectomy as a safe and effective therapeutic option for large, symptomatic splenic pseudocysts, particularly when guided by a multidisciplinary approach and appropriate perioperative planning.
